# Comparison between Arm Port and Chest Port for Optimal Vascular Access Port in Patients with Breast Cancer: A Systematic Review and Meta-Analysis

**DOI:** 10.1155/2020/9082924

**Published:** 2020-02-13

**Authors:** Ye Liu, Li-li Li, Lei Xu, Dong-dong Feng, Yu Cao, Xiao-yun Mao, Jin Zheng, Feng Jin, Bo Chen

**Affiliations:** ^1^Department of Breast Surgery, The First Affiliated Hospital of China Medical University, Shenyang, Liaoning 110001, China; ^2^Department of Urology, The First Affiliated Hospital of China Medical University, Shenyang, Liaoning 110001, China

## Abstract

**Objectives:**

This meta-analysis was conducted to compare the complication rates between arm and chest ports in patients with breast cancer. *Design and Data Sources*. PubMed, Embase, Cochrane library, Chinese National Knowledge Infrastructure (CNKI), and Wanfang database were used to perform a systematic review and meta-analysis of publications published from the inception of the database to 11, October 2019. Our search generated a total of 22 articles published from 2011 to 2019, including 6 comparative studies and 16 single-arm articles, involving 4131 cases and 5272 controls. Single-arm studies combined with comparative studies were also pooled and analyzed. Finally, subgroup analysis was performed to compare the rates of infection and thrombosis between these two ports. *Eligibility Criteria*. Included articles were research studies comparing complication rates of arm ports with chest ports in patients with breast cancer. Any review or meta-analysis article would be removed. *Data Extraction and Synthesis*. Demographic data and information for the following analysis were extracted. DerSimonian and Laird random effect meta-analysis was conducted to analyze comparative studies while Begg's and Egger's tests were used for assessment of publication bias. Meta-regression analysis was performed to explain the sources of heterogeneity.

**Results:**

There was no difference in the risk of overall complications between arm and chest ports for comparative studies (*P*=0.083). While results of pooled comparative and single-arm studies indicated that arm port would increase the overall complication risks with *RR* of 2.64, results of the subgroup analysis showed that there was no difference in the risk of catheter-related infection between these two ports. However, arm port might be associated with the higher thrombosis rates compared with chest port according to the results of the analysis for only comparative studies (*RR* of 2.64, results of the subgroup analysis showed that there was no difference in the risk of catheter-related infection between these two ports. However, arm port might be associated with the higher thrombosis rates compared with chest port according to the results of the analysis for only comparative studies (*P*=0.083). While results of pooled comparative and single-arm studies indicated that arm port would increase the overall complication risks with *RR* of 2.64, results of the subgroup analysis showed that there was no difference in the risk of catheter-related infection between these two ports. However, arm port might be associated with the higher thrombosis rates compared with chest port according to the results of the analysis for only comparative studies (*P*=0.083). While results of pooled comparative and single-arm studies indicated that arm port would increase the overall complication risks with

**Conclusions:**

This study indicated that the arm port might increase the risk of overall complication risks as well as the risk of catheter-related thrombosis compared with the chest port. However, these reported findings still need to be verified by large randomized clinical trials.

## 1. Introduction

Totally implanted venous access port (TIVAP) has been established as an effective and safe procedure for cancer patients, which could be applied to chemotherapy infusion, blood sampling, as well as nutrition supply [1, 2]. TIVAPs have manifested evident superiority with fewer complications and more convenient catheter management compared with peripherally inserted central catheter or any other externally tunneled catheter [3]. In recent years, TIVAPs have been widely used in patients with malignancies needing to receive a long-term catheterization. In general, TIVAPs are inserted into the chest through a subclavian vein or jugular vein under the guidance of ultrasound or surgery. However, with high pneumothorax rate and poor aesthetic appearance, the chest port is being replaced by the peripheral arm port, which is mainly inserted through the basilic vein, and less often the brachial vein. Arm ports are usually implanted via peripheral arm veins at the bedside without using an operating room. Consequently, this access method is safer, has a lower infection rate, is more cost-effective, and has less risk of pneumothorax when compared with the chest ports.

Different implantation methods may lead to different risks of complication occurrence between arm ports and chest ports, which may have a vital role in the catheters span and the safety as well as the quality of life in cancer patients. Based on the guidelines for the prevention of catheter-related infections reported by O'Grady et al., catheter-infections are the most common ones among all catheter-related complications [4]. In addition, venous thrombosis is another catheter-related complication that should not be neglected. Although the arm port contains an obvious superiority over the chest port due to the lower risk of pneumothorax during the perioperative period, it is still controversial due to the incidence rate of late complications. This especially relates to the risk of thrombosis occurrence due to the long length of arm port catheters and the higher movement of the arm part. Previous studies have reported no significant differences in catheter durability and complication risk between chest ports and arm ports [5]; however, these conclusions were not consistent across all studies [6]. Patients can choose between these two venous ports; however, the risk of complications incidence must be considered before implantation. Nonetheless, currently there are no systematic studies concerning the differences of complication rates between these two port systems in patients with breast cancers.

The aim of this meta-analysis was to systematically compare the complication rates of arm ports with chest ports in patients with breast cancer so as to fill in the gaps and provide guidance that would aid decision-making for access ports selection.

## 2. Materials and Methods

### 2.1. Search Strategy

This analysis was reported according to the Preferred Reporting Items for Systematic Reviews and Meta-Analyses statement and checklist (PRISMA). We searched PubMed, Embase, Cochrane Library, CNKI, and Wanfang database for relevant publications. The search strategy was as follows: (implanted venous access ports OR peripheral ports OR forearm ports OR upper arm ports OR arm ports OR chest ports OR ports) AND (breast cancer OR mammary cancer), updated to October 11, 2019. Reference lists of reviews and meta-analysis articles were also searched for potentially relevant articles.

### 2.2. Literature Selection

Literature was selected based on a series of inclusion and exclusion criteria. Inclusion criteria were the following: (1) Participants enrolled in the study were patients with breast cancer; (2) the type of catheter studied was arm port or chest port; (3) the number of total participants and the number of complication cases can be calculated or extracted from papers. The exclusion criteria were as follows: (1) Unrelated publications, reviews, and meta-analysis articles; (2) articles without complete information.

### 2.3. Data Extraction and Quality Assessment

Data were extracted from the enrolled publications by two authors (Ye Liu and Lili Li) independently as listed: the first author, publication country and year, participants' age, sample size, the type and number of complication cases, the mean duration of catheter, and the types of the study design. The quality of the included articles was evaluated based on the National Institutes of Health Quality Assessment Tool for Observational Cohort and Cross-Sectional Studies [7].

### 2.4. Statistical Analysis

Two different sets of meta-analytical methods were applied in this study. First, for studies that directly compared these two port types (hereinafter referred to as comparative studies), we performed the DerSimonian and Laird random effects model (REM) meta-analysis. The pooled relative risk (*RR*) with its 95% CI was evaluated. Begg's and Egger's tests were used to evaluate the possibility of publication bias with *P* value less than 0.05. Second, both single-arm and comparative studies were pooled and synthesized to analyze the difference in complication rates between these two ports. For both analysis models, the heterogeneity among the selected articles was assessed by *Q* test and *I*^2^ value [8]. *I*^2^ ≥ 50% or *P* value for the *Q* test less than 0.05 indicated that there was significant heterogeneity among these studies, and then a meta-regression analysis (grouped by age: <50, ≥50 and not accessible; gender: only female, female and male as well as not accessible; ethnicity: Asian and Caucasian; catheter size: <5F, ≥ 5F and not accessible; quality of the study: fair and good) was used to identify the potential sources of the heterogeneity. The comparison between single-arm studies and single chest studies was conducted by SPSS 22.0 (SPSS Inc., Chicago, IL, USA) and all other statistical analyses were performed by STATA 11.0 (STATA-Corp, College Station, TX, USA) software; the statistical significance was set as *P* < 0.05.

## 3. Results

### 3.1. Publication Search and Studies' Characteristics

**Figure 1:**
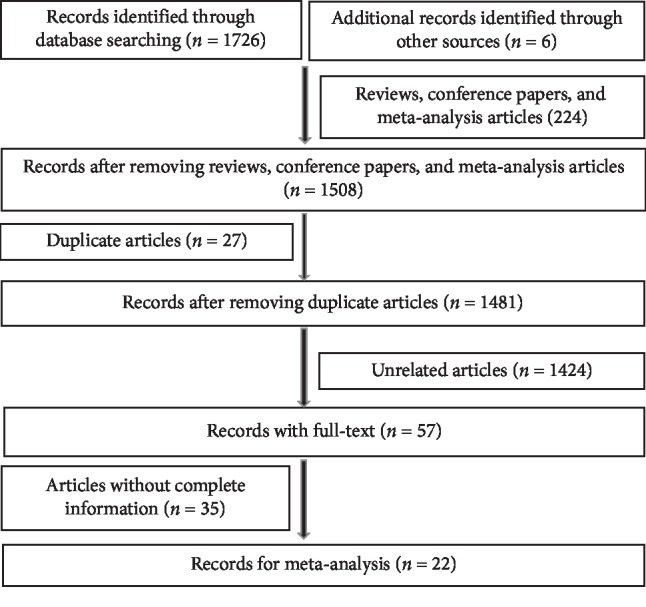
Flow diagram of the study selection for this meta-analysis.

As shown in Figure 1, based on the primary protocol of the publication search, 1732 eligible articles were enrolled, of which 224 articles were excluded since they were reviews, meta-analysis articles, letters, and conference papers. Other 27 duplicated articles and 1424 unrelated articles were also excluded, resulting in 57 papers with full texts. Further, another 35 articles were removed due to missing data. Ultimately, 22 articles published from 2011 to 2019 including 6 comparative studies and 16 single-arm articles (3 articles for arm port and 13 articles for chest port), involving in 4131 cases and 5272 controls [9–30] were included in the study. The main characteristics of these 22 studies are listed in Table 1 and their quality resulted to be generally good as shown in Table 1 and supplementary [Supplementary-material supplementary-material-1].

### 3.2. Pooled Analysis

#### 3.2.1. Meta-Analysis of Comparative Studies

**Figure 2:**
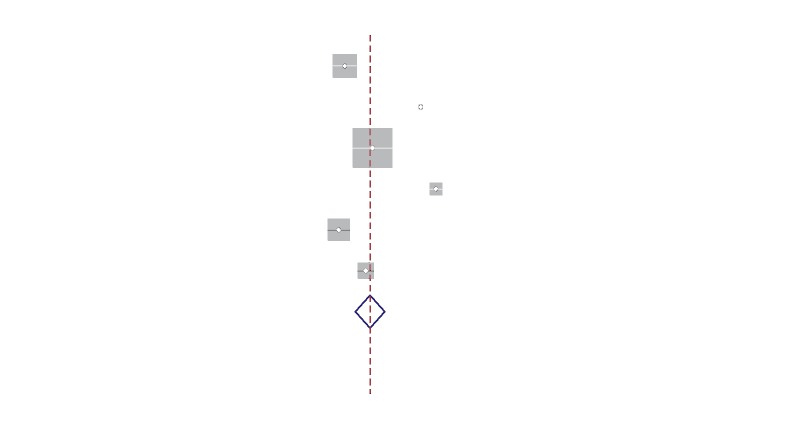
Forest plot of comparative studies' meta-analysis for the association between arm or chest ports and the risk of complication incidence. Each study was shown by a square with its 95% confidence interval shown by the error bars.

**Figure 3:**
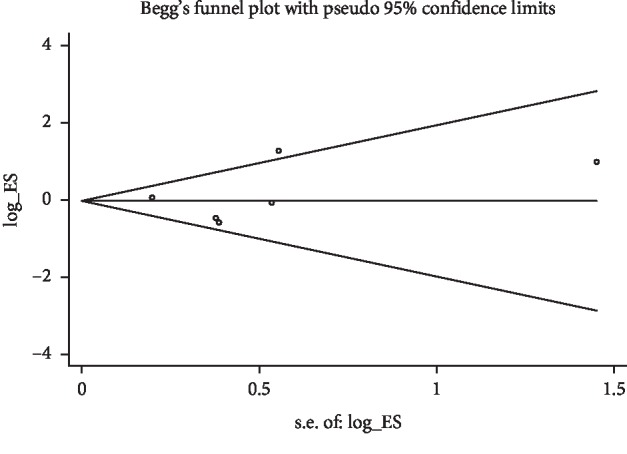
The Begg's funnel plot to assess the publication bias for comparative studies.

Fixed-effect model was performed since no significant heterogeneity was observed among these six comparative studies (*I*^2^ = 48.6%, *P*=0.083). The pooled *RR* was 1.01 (95% CI: 0.77–1.34) with *Z* = 0.09 and *P*=0.928 for arm port versus chest port as shown in Figure 2, which indicated that arm port would not increase the risk of complications compared with the chest port. Publication bias was estimated under a random effect model and no obvious publication bias was found among these studies with *P* value of the Egger's test equal to 0.677 (Figure 3).

#### 3.2.2. Meta-Analysis Combined Single-Arm and Comparative Studies

**Figure 4:**
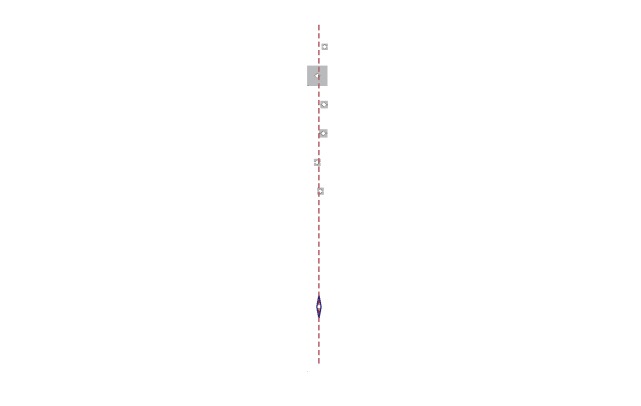
Forest plot of the pooled absolute risk and its 95% CI for arm ports.

**Figure 5:**
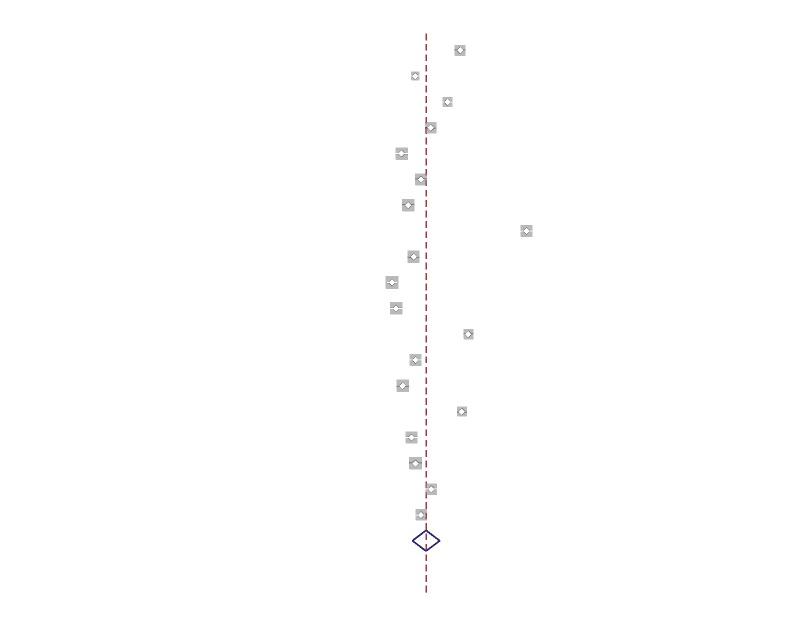
Forest plot of the pooled absolute risk and its 95% CI for chest ports.

The pooled absolute risk (AR) and its 95% CI were 0.06 (0.05, 0.08) for arm port (Figure 4) and 0.09 (0.06, 0.12) for chest port (Figure 5), respectively, combining comparative studies and single-arm studies. The pooled RR and its 95% CI were 2.64 and 2.28–3.07, respectively, indicating a 2.64-fold complication risk of arm port compared with chest port with *P* < 0.001. RD was -0.03 with 95% CI equaling to −0.07–0.02. Since there was high heterogeneity among the studies for chest port, a meta-regression was conducted and no sources of the heterogeneity were found.

In brief, the meta-analysis for the six comparative studies demonstrated that the arm port did not decrease the complication risk compared with the chest port, while a higher risk was found for arm port when combined with the comparative studies and single-arm studies.

### 3.3. Subgroup Analysis

#### 3.3.1. The Rate of Catheter-Related Infection

A total of 18 studies reported the incidence rate of catheter-related infection, of which 4 studies contained a direct comparison of arm port and chest port, whereas the remaining 14 articles were single-arm studies (Table 2). Since no significant heterogeneity existed among these 4 comparative studies (*I*^2^ = 0.0%, *P*=0.776), a fixed-effect model was performed. The pooled RR was 0.58 (95% CI: 0.32–1.06) with *Z* = 1.78 and *P*=0.074 for arm port versus chest port as shown in Supplementary [Supplementary-material supplementary-material-1], indicating that there was no statistical difference of the complication risk between these two ports. The same result was obtained when summarizing and analyzing all of the studies (*RR*: 1.63; 95% CI: 0.97–2.75; *P*=0.064).

#### 3.3.2. Rate of Catheter-Related Thrombosis

A total of 19 studies reported the occurrence rate for catheter-related thrombosis, including 4 comparative studies and 15 single-arm studies (Table 2). Since no significant heterogeneity was found among these 4 comparative studies (*I*^2^ = 46.1%, *P*=0.135), a fixed-effect model was performed. The pooled *RR* was 2.23 (95% CI: 1.04–4.79) with *Z* = 2.05 and *P*=0.041 for arm port versus chest port, respectively, as shown in Supplementary [Supplementary-material supplementary-material-1], which indicated that arm port would increase the risk of catheter thrombosis compared with chest port. Similar results were also obtained when enrolling the uncontrolled studies (RR: 1.21; 95% CI: 1.02–1.43; *P*=0.029).

## 4. Discussion

TIVAP has a pivotal role in the treatment of patients with malignancy during chemotherapy with a lower rate of infectious complications than other externally tunneled catheters. Although chest ports inserted via internal jugular vein or subclavian vein are considered optimal, other insertion sites such as upper arm to decrease catheter-related complications have also been explored [31, 32]. Peripheral arm ports are comparable to the chest ports in arterial injury and have a prominent advantage of less pneumothorax [33]. Due to safety and cost-effectiveness related to bedside insertion, arm ports have been increasingly applied over recent years. Yet, a comparison of complication rates in patients with breast cancer between the arm port and chest port has not been fully taken into consideration. Thus in this study, we investigated whether arm port could decrease the risk of incidence and development of catheter-related complications in patients with breast cancer compared with chest ports. To our knowledge, this was the first systematic review and meta-analysis that assessed and compared the incidence rate of complications between arm ports and chest ports in patients with breast cancer.

Overall, in the present study, no difference of the complication risks was found between arm and chest ports from pooled estimates for comparative studies. However, results of pooled comparative and single-arm studies indicated that the overall complication risks for arm port were 2.64 times as much as risks for chest port, showing a prominent superiority for chest ports with less risks of complication incidence. In the subgroup analysis for catheter-related infection, arm port might not increase the infection risk compared with chest port. Besides catheter-related infection, catheter-related thrombosis is another particular concern because the use of TIVAPs in malignant diseases has been related to a higher thrombosis incidence risk. According to the results of this meta-analysis, arm port might be associated with the higher thrombosis rates compared with chest port for comparative studies (RR = 2.23, *P*=0.041) as well as pooled comparative and single-arm studies (RR = 1.21, *P*=0.029). Above all, although there was no association between the placement of TIVAPs via arm port and the high risk of catheter-related infections, this venous port would increase the risks of overall complications as well as the catheter-related thrombosis compared with chest port. Therefore, it is suggested that chest port should be taken as the primary way to construct a totally implanted venous access port for patients with breast cancer to reduce the risk of catheter-related thrombosis as well as other complications except for the infections.

The present study has some limitations. First, this study might suffer from patient selection bias since most of the enrolled studies were retrospective designs. Furthermore, most of the articles were uncontrolled studies, which might also affect the quality of the pooled estimates. Therefore, prospective randomized controlled studies are warranted to confirm our results that were attained through pooled analysis of 22 articles conducted in varied settings, countries as well as protocols.

## 5. Conclusion

Overall, there was no association between the arm port and the high risk of catheter-related infections; this venous port would increase the risks of overall complications as well as the catheter-related thrombosis compared with chest port. We considered the chest port might be more suitable for patients with breast cancers. However, these results still need to be verified by future studies.

## Figures and Tables

**Table 1:** The main features of included studies for meta-analysis.

Study	Year	Country	Age	Sex	Case (arm)		Control (chest)		Mean duration (days)		Catheter size	Quality of studies
					*N*	*m*	*N*	*m*	Arm	Chest		
Marcy	2005	France	55.7	F	100	10	100	16	168	228	7F	Good
Peynircioglu	2007	Turkey	48.3	F	1	0	15	1	NA	462	5.8F^1^/6.8F^2^	Fair
Dong	2016	China	NA	F	810	45	995	52	NA	NA	NA	Good
Tippit	2018	USA	55	F	147	14	150	4	669.4	512.3	5.5F	Good
Yang	2018	Korea	51.6	F	176	16	55	9	175.2	202.4	5F^1^/8F^2^	Good
Wang	2019	China	52.3	F	95	7	76	6	176	109	4.8F^1^/6.6F^2^	Good
Xu	2017	China	50.6	F	25	0			56		NA	Fair
Decousus	2018	France	63	NA	2664	275			NA		NA	Fair
Awan	2019	Canada	NA	NA	73	4			NA		NA	Good
Pardo	2011	USA	52	NA			45	6		NA	NA	Fair
Teichgraber	2013	Germany	NA	NA			121	12		223	NA	Fair
Piran	2014	Canada	58.0^*∗*^	F/M			183	7		360	6–8F	Fair
Song	2015	Korea	51.4	F			191	15		368	8F	Good
Liu	2017	China	48.7	F			755	225		147.3	7-8F	Good
Mao	2017	China	46.0^*∗*^	F			158	10		245.2	7-8F	Good
Mo	2017	China	NA	F/M			658	12		NA	7-8F	Fair
LeVasseur	2018	Canada	55	NA			62	11		NA	NA	Fair
Song	2018	Korea	51.5	F			209	14		334.6	6.5–8F	Good
Makary	2018	USA	53.1	F			396	16		NA	8F	Fair
Erhancil	2019	Turkey	54	F			68	4		969.8	NA	Good
Isom	2019	USA	52.9	F			581	36		NA	NA	Fair
Zhang	2019	China	52	NA			110	11		NA	NA	Fair

Note. *N*: the number of total participants; m: the number of all patients who suffered from complications; ^*∗*^meant the age was median while others were mean; 1: catheter size for arm port; 2: catheter size for chest port; NA: not accessible.

**Table 2:** The subgroup analysis for the selected studies.

Subgroups	No. of studies	AR [95%CI]	RR [95%CI]	RD [95%CI]	*P*
Infection					
Comparative	4		0.58[0.32–1.055]		0.074
Arm	5	0.027[0.003–0.051]	1.63[0.97–2.75]	0.02[−0.008–0.049]	0.064
Chest	13	0.007[0.002–0.011]			
Thrombosis					
Comparative	4		2.23[1.04–4.79]		0.041
Arm	6	0.045[−0.002–0.093]	1.21[1.02–1.43]	0.003[−0.059–0.065]	0.029
Chest	13	0.042[0.028–0.057]			

AR: absolute risk; RR: risk ratio; RD: risk difference.

## Additional Points

*Strengths and Limitations of This Study*. (1) This meta-analysis study is significant in terms of the arm port and chest port in patients with breast cancer. (2) It provides guidance for decision-making of access ports selection. (3) Most of the enrolled studies were of retrospective design. (4) There was high heterogeneity among different studies.

## Supplementary Materials

Supplementary Materials Figure S1: forest plots of comparative studies' meta-analysis for the association between arm or chest ports and the risk of catheter-related infections. Each study is shown by a square with its 95% Confidence interval shown by the error bars. Figure S2: forest plots of comparative studies' meta-analysis for the association between arm or chest ports and the risk of catheter-related thrombosis. Each study is shown by a square with its 95% Confidence interval shown by the error bars. The Quality Assessment for the Enrolled Studies was included in the Supplementary file S1. Supplementary file S2 was the PRISMA Checklist for this meta-analysis. In addition, forest plots of comparative studies' meta-analysis for the association between arm or chest ports and the risk of catheter-related infections as well as thrombosis were included in the Supplementary figures.
