# Supplementary material for: Comparison between Arm Port and Chest Port for Optimal Vascular Access Port in Patients with Breast Cancer: A Systematic Review and Meta-Analysis
Source: Biomed Res Int. 2020 Feb 13;2020:9082924. doi: 10.1155/2020/9082924 (PMC7040392; doi:10.1155/2020/9082924)
Supplement: Supplementary Materials — Figure S1: forest plots of comparative studies' meta-analysis for the association between arm or chest ports and the risk of catheter-related infections. Each study is shown by a square with its 95% Confidence interval shown by the error bars. Figure S2: forest plots of comparative studies' meta-analysis for the association between arm or chest ports and the risk of catheter-related thrombosis. Each study is shown by a square with its 95% Confidence interval shown by the error bars. The Quality Assessment for the Enrolled Studies was included in the Supplementary file S1. Supplementary file S2 was the PRISMA Checklist for this meta-analysis. In addition, forest plots of comparative studies' meta-analysis for the association between arm or chest ports and the risk of catheter-related infections as well as thrombosis were included in the Supplementary figures. [file 9082924.f1.zip › Supplementary figures.doc]

**Fig.S1** Forest plots of comparative studies’ meta-analysis for the association between arm or chest ports and the risk of catheter-related infections. Each study is shown by square with its 95% Confidence interval showed by the error bars.

**Fig.S2** Forest plots of comparative studies’ meta-analysis for the association between arm or chest ports and the risk of catheter-related thrombosis. Each study is shown by square with its 95% Confidence interval showed by the error bars.
