# Supplementary material for: Comparison between Arm Port and Chest Port for Optimal Vascular Access Port in Patients with Breast Cancer: A Systematic Review and Meta-Analysis
Source: Biomed Res Int. 2020 Feb 13;2020:9082924. doi: 10.1155/2020/9082924 (PMC7040392; doi:10.1155/2020/9082924)
Supplement: Supplementary Materials — Figure S1: forest plots of comparative studies' meta-analysis for the association between arm or chest ports and the risk of catheter-related infections. Each study is shown by a square with its 95% Confidence interval shown by the error bars. Figure S2: forest plots of comparative studies' meta-analysis for the association between arm or chest ports and the risk of catheter-related thrombosis. Each study is shown by a square with its 95% Confidence interval shown by the error bars. The Quality Assessment for the Enrolled Studies was included in the Supplementary file S1. Supplementary file S2 was the PRISMA Checklist for this meta-analysis. In addition, forest plots of comparative studies' meta-analysis for the association between arm or chest ports and the risk of catheter-related infections as well as thrombosis were included in the Supplementary figures. [file 9082924.f1.zip › Supplementary file S1.doc]

| Study/criteria |  | 1 | 2 | 3 | 4 | 5 | 6 | 7 | 8 | 9 | 10 | 11 | 12 | 13 | 14 | Quality rating |
| --- | --- | --- | --- | --- | --- | --- | --- | --- | --- | --- | --- | --- | --- | --- | --- | --- |
| SS Yang \| | 2018 | Y | Y | Y | Y | N | Y | Y | N | Y | N | Y | N | Y | Y | good |
| D Tippit \| | 2018 | Y | Y | Y | Y | N | Y | Y | N | Y | N | Y | N | Y | Y | good |
| MS Makary \| | 2007 | Y | Y | Y | Y | N | Y | NR | N | Y | N | Y | N | Y | N | fair |
| LeVasseur \| | 2005 | Y | Y | Y | Y | N | Y | NR | N | Y | N | Y | NR | Y | N | fair |
| H Liu \| | 2017 | Y | Y | Y | Y | N | Y | Y | N | Y | N | Y | N | Y | N | good |
| XF Mo \| | 2018 | Y | Y | Y | Y | N | Y | NR | N | Y | N | Y | N | Y | N | fair |
| HP Xu | 2018 | Y | Y | Y | Y | N | Y | N | N | Y | N | Y | N | Y | N | fair |
| L Mao \| | 2017 | Y | Y | Y | Y | N | Y | Y | N | Y | N | Y | N | Y | N | good |
| MG Song \| | 2017 | Y | Y | Y | Y | N | Y | Y | N | Y | N | Y | N | Y | N | good |
| S Piran \| | 2017 | Y | N | Y | Y | N | Y | Y | N | Y | N | Y | N | Y | N | fair |
| U Teichgraber \| | 2015 | Y | N | Y | Y | N | Y | NR | N | Y | N | Y | N | Y | N | fair |
| I Pardo \| | 2014 | Y | N | Y | Y | N | Y | NR | N | Y | N | Y | N | Y | N | fair |
| Peynircioglu \| | 2013 | Y | N | Y | Y | N | Y | NR | N | Y | N | Y | N | Y | Y | fair |
| PY Marcy \| | 2011 | Y | Y | Y | Y | N | Y | Y | N | Y | N | Y | N | Y | Y | good |
| AA Awan | 2019 | Y | Y | Y | Y | N | Y | NR | N | Y | N | Y | Y | Y | Y | good |
| B Erhancil | 2019 | Y | Y | Y | Y | N | Y | Y | N | Y | N | Y | N | Y | N | good |
| H Decousus | 2018 | Y | Y | Y | Y | N | Y | NR | N | Y | N | Y | N | Y | N | fair |
| MG Song | 2018 | Y | Y | Y | Y | N | Y | Y | N | Y | N | Y | N | Y | N | good |
| C Isom | 2019 | Y | Y | Y | Y | N | Y | NR | N | Y | N | Y | N | Y | N | fair |
| P Zhang | 2019 | Y | Y | Y | Y | N | Y | NR | N | Y | N | Y | N | Y | N | fair |
| J Dong | 2016 | Y | Y | Y | Y | N | Y | NR | N | Y | N | Y | N | Y | Y | good |
| XY Wang | 2019 | Y | Y | Y | Y | N | Y | Y | N | Y | N | Y | N | Y | Y | good |

**Quality Assessment for the Enrolled Studies**

Quality Assessment Tool for Observational Cohort and Cross-Sectional Studies

| **Criteria** | **Yes** | **No** | **Other (CD, NR, NA)*** |
| --- | --- | --- | --- |
| 1. Was the research question or objective in this paper clearly stated? |  |  |  |
| 2. Was the study population clearly specified and defined? |  |  |  |
| 3. Was the participation rate of eligible persons at least 50%? |  |  |  |
| 4. Were all the subjects selected or recruited from the same or similar populations (including the same time period)? Were inclusion and exclusion criteria for being in the study prespecified and applied uniformly to all participants? |  |  |  |
| 5. Was a sample size justification, power description, or variance and effect estimates provided? |  |  |  |
| 6. For the analyses in this paper, were the exposure(s) of interest measured prior to the outcome(s) being measured? |  |  |  |
| 7. Was the timeframe sufficient so that one could reasonably expect to see an association between exposure and outcome if it existed? |  |  |  |
| 8. For exposures that can vary in amount or level, did the study examine different levels of the exposure as related to the outcome (e.g., categories of exposure, or exposure measured as continuous variable)? |  |  |  |
| 9. Were the exposure measures (independent variables) clearly defined, valid, reliable, and implemented consistently across all study participants? |  |  |  |
| 10. Was the exposure(s) assessed more than once over time? |  |  |  |
| 11. Were the outcome measures (dependent variables) clearly defined, valid, reliable, and implemented consistently across all study participants? |  |  |  |
| 12. Were the outcome assessors blinded to the exposure status of participants? |  |  |  |
| 13. Was loss to follow-up after baseline 20% or less? |  |  |  |
| 14. Were key potential confounding variables measured and adjusted statistically for their impact on the relationship between exposure(s) and outcome(s)? |  |  |  |

**Quality Rating (Good, Fair, or Poor)**

Rater #1 initials:

Rater #2 initials:

Additional Comments (If POOR, please state why):

*CD, cannot determine; NA, not applicable; NR, not reported

**Guidance for Assessing the Quality of Observational Cohort and Cross-Sectional Studies**

The guidance document below is organized by question number from the tool for quality assessment of observational cohort and cross-sectional studies.

**Question 1. Research question**

Did the authors describe their goal in conducting this research? Is it easy to understand what they were looking to find? This issue is important for any scientific paper of any type. Higher quality scientific research explicitly defines a research question.

**Questions 2 and 3. Study population**

Did the authors describe the group of people from which the study participants were selected or recruited, using demographics, location, and time period? If you were to conduct this study again, would you know who to recruit, from where, and from what time period? Is the cohort population free of the outcomes of interest at the time they were recruited?

An example would be men over 40 years old with type 2 diabetes who began seeking medical care at Phoenix Good Samaritan Hospital between January 1, 1990 and December 31, 1994. In this example, the population is clearly described as: (1) who (men over 40 years old with type 2 diabetes); (2) where (Phoenix Good Samaritan Hospital); and (3) when (between January 1, 1990 and December 31, 1994). Another example is women ages 34 to 59 years of age in 1980 who were in the nursing profession and had no known coronary disease, stroke, cancer, hypercholesterolemia, or diabetes, and were recruited from the 11 most populous States, with contact information obtained from State nursing boards.

In cohort studies, it is crucial that the population at baseline is free of the outcome of interest. For example, the nurses' population above would be an appropriate group in which to study incident coronary disease. This information is usually found either in descriptions of population recruitment, definitions of variables, or inclusion/exclusion criteria.

You may need to look at prior papers on methods in order to make the assessment for this question. Those papers are usually in the reference list.

If fewer than 50% of eligible persons participated in the study, then there is concern that the study population does not adequately represent the target population. This increases the risk of bias.

**Question 4. Groups recruited from the same population and uniform eligibility criteria**

Were the inclusion and exclusion criteria developed prior to recruitment or selection of the study population? Were the same underlying criteria used for all of the subjects involved? This issue is related to the description of the study population, above, and you may find the information for both of these questions in the same section of the paper.

Most cohort studies begin with the selection of the cohort; participants in this cohort are then measured or evaluated to determine their exposure status. However, some cohort studies may recruit or select exposed participants in a different time or place than unexposed participants, especially retrospective cohort studies–which is when data are obtained from the past (retrospectively), but the analysis examines exposures prior to outcomes. For example, one research question could be whether diabetic men with clinical depression are at higher risk for cardiovascular disease than those without clinical depression. So, diabetic men with depression might be selected from a mental health clinic, while diabetic men without depression might be selected from an internal medicine or endocrinology clinic. This study recruits groups from different clinic populations, so this example would get a "no."

However, the women nurses described in the question above were selected based on the same inclusion/exclusion criteria, so that example would get a "yes."

**Question 5. Sample size justification**

Did the authors present their reasons for selecting or recruiting the number of people included or analyzed? Do they note or discuss the statistical power of the study? This question is about whether or not the study had enough participants to detect an association if one truly existed.

A paragraph in the methods section of the article may explain the sample size needed to detect a hypothesized difference in outcomes. You may also find a discussion of power in the discussion section (such as the study had 85 percent power to detect a 20 percent increase in the rate of an outcome of interest, with a 2-sided alpha of 0.05). Sometimes estimates of variance and/or estimates of effect size are given, instead of sample size calculations. In any of these cases, the answer would be "yes."

However, observational cohort studies often do not report anything about power or sample sizes because the analyses are exploratory in nature. In this case, the answer would be "no." This is not a "fatal flaw." It just may indicate that attention was not paid to whether the study was sufficiently sized to answer a prespecified question–i.e., it may have been an exploratory, hypothesis-generating study.

**Question 6. Exposure assessed prior to outcome measurement**

This question is important because, in order to determine whether an exposure causes an outcome, the exposure must come before the outcome.

For some prospective cohort studies, the investigator enrolls the cohort and then determines the exposure status of various members of the cohort (large epidemiological studies like Framingham used this approach). However, for other cohort studies, the cohort is selected based on its exposure status, as in the example above of depressed diabetic men (the exposure being depression). Other examples include a cohort identified by its exposure to fluoridated drinking water and then compared to a cohort living in an area without fluoridated water, or a cohort of military personnel exposed to combat in the Gulf War compared to a cohort of military personnel not deployed in a combat zone.

With either of these types of cohort studies, the cohort is followed forward in time (i.e., prospectively) to assess the outcomes that occurred in the exposed members compared to nonexposed members of the cohort. Therefore, you begin the study in the present by looking at groups that were exposed (or not) to some biological or behavioral factor, intervention, etc., and then you follow them forward in time to examine outcomes. If a cohort study is conducted properly, the answer to this question should be "yes," since the exposure status of members of the cohort was determined at the beginning of the study before the outcomes occurred.

For retrospective cohort studies, the same principal applies. The difference is that, rather than identifying a cohort in the present and following them forward in time, the investigators go back in time (i.e., retrospectively) and select a cohort based on their exposure status in the past and then follow them forward to assess the outcomes that occurred in the exposed and nonexposed cohort members. Because in retrospective cohort studies the exposure and outcomes may have already occurred (it depends on how long they follow the cohort), it is important to make sure that the exposure preceded the outcome.

Sometimes cross-sectional studies are conducted (or cross-sectional analyses of cohort-study data), where the exposures and outcomes are measured during the same timeframe. As a result, cross-sectional analyses provide weaker evidence than regular cohort studies regarding a potential causal relationship between exposures and outcomes. For cross-sectional analyses, the answer to Question 6 should be "no."

**Question 7. Sufficient timeframe to see an effect**

Did the study allow enough time for a sufficient number of outcomes to occur or be observed, or enough time for an exposure to have a biological effect on an outcome? In the examples given above, if clinical depression has a biological effect on increasing risk for CVD, such an effect may take years. In the other example, if higher dietary sodium increases BP, a short timeframe may be sufficient to assess its association with BP, but a longer timeframe would be needed to examine its association with heart attacks.

The issue of timeframe is important to enable meaningful analysis of the relationships between exposures and outcomes to be conducted. This often requires at least several years, especially when looking at health outcomes, but it depends on the research question and outcomes being examined.

Cross-sectional analyses allow no time to see an effect, since the exposures and outcomes are assessed at the same time, so those would get a "no" response.

**Question 8. Different levels of the exposure of interest**

If the exposure can be defined as a range (examples: drug dosage, amount of physical activity, amount of sodium consumed), were multiple categories of that exposure assessed? (for example, for drugs: not on the medication, on a low dose, medium dose, high dose; for dietary sodium, higher than average U.S. consumption, lower than recommended consumption, between the two). Sometimes discrete categories of exposure are not used, but instead exposures are measured as continuous variables (for example, mg/day of dietary sodium or BP values).

In any case, studying different levels of exposure (where possible) enables investigators to assess trends or dose-response relationships between exposures and outcomes–e.g., the higher the exposure, the greater the rate of the health outcome. The presence of trends or dose-response relationships lends credibility to the hypothesis of causality between exposure and outcome.

For some exposures, however, this question may not be applicable (e.g., the exposure may be a dichotomous variable like living in a rural setting versus an urban setting, or vaccinated/not vaccinated with a one-time vaccine). If there are only two possible exposures (yes/no), then this question should be given an "NA," and it should not count negatively towards the quality rating.

**Question 9. Exposure measures and assessment**

Were the exposure measures defined in detail? Were the tools or methods used to measure exposure accurate and reliable–for example, have they been validated or are they objective? This issue is important as it influences confidence in the reported exposures. When exposures are measured with less accuracy or validity, it is harder to see an association between exposure and outcome even if one exists. Also as important is whether the exposures were assessed in the same manner within groups and between groups; if not, bias may result.

For example, retrospective self-report of dietary salt intake is not as valid and reliable as prospectively using a standardized dietary log plus testing participants' urine for sodium content. Another example is measurement of BP, where there may be quite a difference between usual care, where clinicians measure BP however it is done in their practice setting (which can vary considerably), and use of trained BP assessors using standardized equipment (e.g., the same BP device which has been tested and calibrated) and a standardized protocol (e.g., patient is seated for 5 minutes with feet flat on the floor, BP is taken twice in each arm, and all four measurements are averaged). In each of these cases, the former would get a "no" and the latter a "yes."

Here is a final example that illustrates the point about why it is important to assess exposures consistently across all groups: If people with higher BP (exposed cohort) are seen by their providers more frequently than those without elevated BP (nonexposed group), it also increases the chances of detecting and documenting changes in health outcomes, including CVD-related events. Therefore, it may lead to the conclusion that higher BP leads to more CVD events. This may be true, but it could also be due to the fact that the subjects with higher BP were seen more often; thus, more CVD-related events were detected and documented simply because they had more encounters with the health care system. Thus, it could bias the results and lead to an erroneous conclusion.

**Question 10. Repeated exposure assessment**

Was the exposure for each person measured more than once during the course of the study period? Multiple measurements with the same result increase our confidence that the exposure status was correctly classified. Also, multiple measurements enable investigators to look at changes in exposure over time, for example, people who ate high dietary sodium throughout the followup period, compared to those who started out high then reduced their intake, compared to those who ate low sodium throughout. Once again, this may not be applicable in all cases. In many older studies, exposure was measured only at baseline. However, multiple exposure measurements do result in a stronger study design.

**Question 11. Outcome measures**

Were the outcomes defined in detail? Were the tools or methods for measuring outcomes accurate and reliable–for example, have they been validated or are they objective? This issue is important because it influences confidence in the validity of study results. Also important is whether the outcomes were assessed in the same manner within groups and between groups.

An example of an outcome measure that is objective, accurate, and reliable is death–the outcome measured with more accuracy than any other. But even with a measure as objective as death, there can be differences in the accuracy and reliability of how death was assessed by the investigators. Did they base it on an autopsy report, death certificate, death registry, or report from a family member? Another example is a study of whether dietary fat intake is related to blood cholesterol level (cholesterol level being the outcome), and the cholesterol level is measured from fasting blood samples that are all sent to the same laboratory. These examples would get a "yes." An example of a "no" would be self-report by subjects that they had a heart attack, or self-report of how much they weigh (if body weight is the outcome of interest).

Similar to the example in Question 9, results may be biased if one group (e.g., people with high BP) is seen more frequently than another group (people with normal BP) because more frequent encounters with the health care system increases the chances of outcomes being detected and documented.

**Question 12. Blinding of outcome assessors**

Blinding means that outcome assessors did not know whether the participant was exposed or unexposed. It is also sometimes called "masking." The objective is to look for evidence in the article that the person(s) assessing the outcome(s) for the study (for example, examining medical records to determine the outcomes that occurred in the exposed and comparison groups) is masked to the exposure status of the participant. Sometimes the person measuring the exposure is the same person conducting the outcome assessment. In this case, the outcome assessor would most likely not be blinded to exposure status because they also took measurements of exposures. If so, make a note of that in the comments section.

As you assess this criterion, think about whether it is likely that the person(s) doing the outcome assessment would know (or be able to figure out) the exposure status of the study participants. If the answer is no, then blinding is adequate. An example of adequate blinding of the outcome assessors is to create a separate committee, whose members were not involved in the care of the patient and had no information about the study participants' exposure status. The committee would then be provided with copies of participants' medical records, which had been stripped of any potential exposure information or personally identifiable information. The committee would then review the records for prespecified outcomes according to the study protocol. If blinding was not possible, which is sometimes the case, mark "NA" and explain the potential for bias.

**Question 13. Followup rate**

Higher overall followup rates are always better than lower followup rates, even though higher rates are expected in shorter studies, whereas lower overall followup rates are often seen in studies of longer duration. Usually, an acceptable overall followup rate is considered 80 percent or more of participants whose exposures were measured at baseline. However, this is just a general guideline. For example, a 6-month cohort study examining the relationship between dietary sodium intake and BP level may have over 90 percent followup, but a 20-year cohort study examining effects of sodium intake on stroke may have only a 65 percent followup rate.

**Question 14. Statistical analyses**

Were key potential confounding variables measured and adjusted for, such as by statistical adjustment for baseline differences? Logistic regression or other regression methods are often used to account for the influence of variables not of interest.

This is a key issue in cohort studies, because statistical analyses need to control for potential confounders, in contrast to an RCT, where the randomization process controls for potential confounders. All key factors that may be associated both with the exposure of interest and the outcome–that are not of interest to the research question–should be controlled for in the analyses.

For example, in a study of the relationship between cardiorespiratory fitness and CVD events (heart attacks and strokes), the study should control for age, BP, blood cholesterol, and body weight, because all of these factors are associated both with low fitness and with CVD events. Well-done cohort studies control for multiple potential confounders.
